# Condition-Specific Transcriptional and Metabolic Divergence in the Dual-Fungal Symbiosis of JinEr Mushroom Under Postharvest Low-Temperature Stress

**DOI:** 10.3390/genes17030296

**Published:** 2026-02-28

**Authors:** Yuntao Li, Hao Tang, Fuwei Wang, Chaotian Lv, Bin Zhang, Huan Li

**Affiliations:** College of Food and Biological Engineering, Bengbu University, Caoshan Road, Bengbu 233030, China

**Keywords:** response divergence, JinEr mushroom, *Naematelia aurantialba*, *Stereum hirsutum*, postharvest low-temperature stress, integrated multi-omics

## Abstract

Background: The JinEr mushroom results from the heterogeneous symbiosis of *Naematelia aurantialba* and *Stereum hirsutum*, with low-temperature storage being key for postharvest quality preservation. However, the species-specific low-temperature response patterns remain unclear. Methods: An integrated approach combining metabolomics, transcriptomics (dual-genome alignment), and spatially resolved enzyme assays was used to dissect responses at 0 °C and 4 °C. Results: The two fungi displayed distinct stress response tendencies under the studied conditions. *N. aurantialba* showed enhanced stress defense (DNA repair, antioxidant pathways) with defense-related enzyme activities concentrated in its apical/middle enrichment regions. *S. hirsutum* was observed to maintain overall metabolic activity at the pathway level, and its metabolic enzyme activities were predominant in the basal region. The symbiotic system exhibited temperature-dependent plasticity stress responses. Storage at 0 °C induced a survival-oriented response with slower crude polysaccharide degradation. In contrast, storage at 4 °C supported active metabolic defense coordination but more pronounced polysaccharide loss. Conclusions: These observed defense- and metabolism-biased differential responses suggest a cold stress-specific coordination working model within the symbiotic system under postharvest cold stress. A temperature of 0 °C is more suitable for enabling JinEr mushroom postharvest storage to retain polysaccharides. This study advances our understanding of heterogeneous symbiotic fungi’s postharvest biology and provides a temperature-targeted theoretical basis for storage optimization.

## 1. Introduction

Edible fungi are globally consumed for their nutritional, sensory, and medicinal value [1]. Among them, the JinEr mushroom (Golden Ear) holds high economic and functional value in Asian markets, especially China, owing to its unique texture and bioactive components, including polysaccharides [2,3]. A defining feature that distinguishes JinEr from most cultivated mushrooms is that it is not a homogeneous fruiting body derived from a single fungus, but a heterologous symbiotic complex formed by *Naematelia aurantialba* and *Stereum hirsutum* [4]. This symbiosis is essential for the development of commercially valuable fruiting bodies: pure cultures of *N. aurantialba* fail to form fruiting bodies, while *S. hirsutum* alone produces morphologically distinct, non-commercial structures [5]. Recent work has revealed spatial partitioning within the fruiting body, with a balanced *S. hirsutum*:*N. aurantialba* ratio in the apical region and strong dominance by *S. hirsutum* in the basal part and substrate [4]. This spatial separation raises the hypothesis of functional differentiation between the two symbionts. However, the underlying molecular basis for such potential differentiation, especially under environmental stress, remains largely unknown.

As with most horticultural products valued for freshness, JinEr mushrooms face severe postharvest preservation challenges. A distinctive feature that complicates its postharvest biology is that the commercially harvested fruiting body is not a homogeneous fungal tissue, but a heterogeneous symbiotic complex of *N. aurantialba* and *S. hirsutum*. Low-temperature storage is among the most widely applied and effective approaches to retarding senescence and maintaining nutritional and sensory quality in perishable edible fungi [6,7]. The biological response to low temperatures is complex and temperature-dependent. Studies on homogeneous, single-species cultivated mushrooms have demonstrated that even small temperature differences within the low-temperature range can strongly influence postharvest shelf life and quality [5]. A sound, mechanistic understanding of the underlying molecular pathways is therefore critical for optimizing storage protocols. Extensive research has investigated low-temperature storage physiology in numerous fruits and vegetables, as well as in several homogeneous cultivated mushrooms [8,9,10]. However, the molecular response mechanisms of the heterogeneous, symbiotic JinEr mushroom system remain largely uncharacterized. Critically, it remains poorly understood how the two fungal partners—*N. aurantialba* and *S. hirsutum*—that together form the JinEr fruiting body coordinate their responses to postharvest cold stress, despite their distinct spatial distribution and presumptive functional roles. Addressing this knowledge gap is essential for advancing the postharvest biology of this one-of-a-kind symbiotic edible fungus.

Therefore, this study employs an integrated systems biology approach, combining untargeted metabolomics and transcriptomics, to investigate the dynamic physiological changes in JinEr fruiting body during storage under two low-temperature conditions (0 °C and 4 °C). The novel objective is to move beyond viewing JinEr mushroom as a single entity. Instead, the focus is to explore the respective transcriptional and metabolic adjustments of *N. aurantialba* and *S. hirsutum* under low-temperature stress, and to propose a context-specific response model to examine how these changes may relate to the overall preservation outcome of the JinEr mushroom complex.

## 2. Materials and Methods

### 2.1. Biological Material, Storage Treatments, and Sampling

The fresh fruiting bodies of JinEr mushroom, a heterogeneous complex formed by *N. aurantialba* and *S. hirsutum*, were procured from a single cultivation batch from Anhui Fengyuan Agricultural Development Co., Ltd. (Bengbu, Anhui, China) to minimize biological variation. Upon arrival, we selected fruiting bodies displaying uniform size, color, and maturity without physical damage. Samples were divided into five groups (3 biological replicates per group, 15 total): OS (Day 0 control, no cold storage, direct analysis); LS1/LS2 (0 °C storage for 4/8 days); HS1/HS2 (4 °C storage for 4/8 days). Immediately after selection, samples were directly placed into climate chambers at the target temperatures. These two temperatures were selected for practical and biological relevance: 4 °C is the standard industrial storage temperature for edible fungi, while 0 °C represents a near-freezing non-frozen condition to compare mild and severe cold stress. Both avoid ice crystal damage to fruiting body tissues. All treatments were conducted in climate-controlled chambers (85–90% relative humidity). Fruiting bodies were placed directly in open trays without packaging, ensuring the gas composition within the chamber remained consistent with that in ambient air. Refrigerated groups were sampled at respective time points. Post-storage, entire fruiting bodies were ground to fine powder in liquid nitrogen, homogenized, and aliquoted for metabolomic and transcriptomic analyses. Samples were labeled with “M” (non-targeted metabolomics, e.g., LSM1) or “T” (transcriptomics, e.g., LST1) for distinction.

### 2.2. Non-Targeted Metabolomic Analysis

Briefly, 100 mg of JinEr powder was homogenized in 1 mL ice-cold 80% methanol, incubated on ice for 5 min, and centrifuged at 15,000× *g* for 20 min at 4 °C. The supernatant was diluted with LC-MS grade water to 53% methanol and recentrifuged similarly. UHPLC-MS/MS analysis was conducted by Novogene Co., Ltd. (Beijing, China) using a Thermo Hypersil GOLD™ C18 column (100 × 2.1 mm, 1.9 μm) at 40 °C. The mobile phase consisted of water (0.1% formic acid, A) and methanol (0.1% formic acid, B) at 0.2 mL/min with the following gradient: 0–1.5 min (2% B), 1.5–4.5 min (linear increase to 85% B), 4.5–14.5 min (100% B), 14.5–14.6 min (return to 2% B), 14.6–16 min (re-equilibration). Mass spectrometry was performed on a Q-Exactive HF-X (Thermo Fisher Scientific, Waltham, MA, USA) in polarity-switching mode, with full-scan at *m*/*z* 100–1500 (resolution 120,000), spray voltage at ±3.5 kV, capillary temperature at 320 °C, sheath gas at 35 arb, and auxiliary gas at 10 L/min (350 °C).

Raw data were processed with XCMS (v3.12.0) for peak detection, alignment, and quantification. Metabolites were identified by matching experimental MS/MS spectra to theoretical spectra in HMDB, LIPIDMAPS, and KEGG databases (mass tolerance ± 10 ppm). After blank subtraction and normalization, compounds with a coefficient of variation (CV) > 30% in quality control (QC) samples were excluded. Multivariate (principal component analysis, PCA) and univariate (*t*-test) analyses were performed with metaX (v1.4.20). Differentially accumulated metabolites (DAMs) were defined as compounds satisfying all criteria: a variable importance in projection (VIP) score from OPLS-DA model > 1, a *p*_adj_ (*t*-test) < 0.05, and a fold change > 1.5 or <0.67. Significantly enriched KEGG pathways were identified via a hypergeometric test (*p*_adi_ < 0.05). The differential abundance score (DAS) is a metric (−1 to 1) that quantifies KEGG pathway trends based on DAM regulation, with extremes indicating full activation or inhibition and intermediate values suggesting partial or no net change. DAS = (increased metabolites − decreased metabolites)/total measured metabolites in the pathway [11].

### 2.3. RNA-Seq Analysis and Dual-Genome Alignment Strategy

Total RNA was extracted from approximately 100 mg of ground JinEr powder using the RNAprep Pure Total RNA Kit (Tiangen, Beijing, China). RNA quality was assessed with a NanoDrop spectrophotometer (Thermo Fisher Scientific, Waltham, MA, USA) and an Agilent 2100 Bioanalyzer (Agilent Technologies, Santa Clara, CA, USA), confirming an RNA Integrity Number (RIN) > 8.0 for all samples. Sequencing libraries were prepared with the NEBNext^®^ Ultra™ RNA Library Prep Kit (New England Biolabs, Ipswich, MA, USA) for Illumina^®^ and sequenced on an Illumina HiSeq 4000 platform (150 bp paired-end, PE150) (Illumina, San Diego, CA, USA). Raw reads were processed with cutadapt (v1.18) to remove adapters and low-quality reads, yielding high-quality clean data without residual contamination.

To accurately quantify species-specific transcripts in this dual-fungal symbiotic complex and resolve ambiguous cross-species read mapping, a species-specific decoy-aware quantification strategy was implemented using Salmon (v1.10.0), a quasi-mapping-based tool for transcript abundance estimation. The mRNA sequences of *N. aurantialba* and *S. hirsutum* were extracted from their respective NCBI-derived genome annotation files using gffread, with corresponding genome assemblies used as templates for accurate transcript extraction. A combined decoy-aware index was constructed by integrating transcript sequences from both fungal species. Transcripts derived from each species were designated as target sequences for that species, while transcripts from the partner species were defined as decoy sequences via the decoy parameter, allowing the probabilistic resolution of cross-species multi-mapping reads. Clean reads from each sample were quantified against the combined index with the validateMappings flag enabled, and transcript-level abundance estimates (TPM and counts) were generated for each species. Sequences with high homology between the two fungi were identified by reciprocal BLAST+ v2.14.0 and excluded from subsequent differential expression analysis to reduce assignment ambiguity.

The transcript abundance matrices were imported into R (v 4.3.1) using the tximport package (v1.28.0; Bioconductor) based on a transcript-to-gene mapping table extracted from the genome annotation files. Low-expression genes were filtered out to reduce noise, and we only retained those with counts ≥ 10 in at least 3 samples. Differential expression analysis between experimental groups was performed separately for each fungal species using DESeq2 (v1.40.0), with genes satisfying |log_2_ (fold change)| ≥ 1.0 and an adjusted *p*-value (FDR) < 0.05 defined as differentially expressed genes (DEGs). Cross-species homologous DEGs (identified via BLAST alignment with E-value < 1 × 10^−10^) were excluded to eliminate potential cross-contamination. KEGG pathway enrichment analysis of DEGs was conducted using clusterProfiler (v4.8.0), with an FDR < 0.05 considered significant.

### 2.4. Determination of Enzyme Activities and Crude Polysaccharide Content

To provide biochemical validation for key inferred pathways, the activities of five key enzymes were measured—specifically, superoxide dismutase (SOD) and glutathione reductase (GR) for antioxidant defense, 8-oxoguanine DNA glycosylase 1 (OGG1) for DNA damage repair, and citrate synthase (CS) and phosphofructokinase (PFK) for energy metabolism. Each of the five storage treatment groups included three biological replicates (i.e., three independent fruiting bodies) and the apical (T), middle (M) and basal (B) regions were sampled separately from each fruiting body for analysis. This sampling strategy was based on the known species-specific spatial distribution of the two fungi in JinEr fruiting bodies [4], where *N. aurantialba* is relatively enriched in the apical and middle regions, and *S. hirsutum* dominates the basal region. All enzymatic assays were conducted using commercial spectrophotometric kits (Soleibao Biotechnology Co., Ltd., Wuhan, China), following the manufacturer’s instructions.

Additionally, the content of crude polysaccharides in JinEr was determined using entire fruiting bodies, with three biological replicates consistent with those in the enzyme activity assays. The determination was performed by spectrophotometry, following the national standard of the Ministry of Agriculture and Rural Affairs of the People’s Republic of China (NY/T 1676-2023), entitled “Determination of crude polysaccharides in edible fungi—Spectrophotometry”.

### 2.5. Key Gene Selection and Expression Analysis

Key DEGs were selected based on the functional emphases of the two symbiotic fungi: *N. aurantialba* in stress defense and *S. hirsutum* in metabolic processes. The selection adhered to three criteria: significant enrichment in KEGG pathways (FDR < 0.05), significant differential expression (|log_2_FC| ≥ 1.0, FDR < 0.05), and functional consistency with the core biological role of each fungus. This resulted in the identification of glutathione S-transferase gene (*Gst*), mutL homolog 1 gene (*Mlh1*), and *Ogg1* from *N. aurantialba*, and *Cs*, malate dehydrogenase gene (*Mdh*), and cysteine synthase gene (*Cysk*) from *S. hirsutum*. To facilitate the direct comparison of expression and abundance patterns across different genes, the quantified levels (FPKM: Fragments Per Kilobase of transcript per Million mapped fragments) were normalized using Z-score standardization.

### 2.6. Gene–Metabolite Correlation Analysis

Potential regulatory interactions between key DEGs and DAMs were investigated using Spearman’s rank correlation analysis. Antioxidant metabolites (L-ascorbic acid, NADPH) were paired with stress-related genes from *N. aurantialba*, while energy and amino acid metabolites (α-ketoglutarate, L-homocysteine) were correlated with metabolic genes from *S. hirsutum*. The analysis was conducted separately for the two temperature regimes (HS: 4 °C; LS: 0 °C). Benjamini–Hochberg (BH) correction was applied to control false positives, with correlations deemed significant at *p*_adj_ < 0.05. Correlation strength was categorized by |r|: weak (0.3–0.5), moderate (0.5–0.8), strong (>0.8).

### 2.7. Data Analysis and Integration

All statistical analyses and data visualizations for enzyme activity and crude polysaccharide content were conducted using R (version 4.3.1). We primarily used the ggplot2 (v3.4.0), dplyr (v1.1.0), rstatix, and ggpubr packages. For each index, the mean value of technical replicates for each biological sample was calculated. Mann–Whitney U tests were applied for targeted pairwise comparisons among the groups. *p*-values from multiple comparison procedures were adjusted using the Benjamini–Hochberg method to control the false discovery rate. All statistical analyses were performed using SPSS software version 24 (IBM, Armonk, NY, USA).

### 2.8. Nucleic Acid Sequence Accession Numbers

The raw sequence data reported in this paper have been deposited in the Genome Sequence Archive in National Genomics Data Center (Nucleic Acids Res 2025), China National Center for Bioinformation/Beijing Institute of Genomics, Chinese Academy of Sciences (GSA: CRA038650). These are publicly accessible at https://ngdc.cncb.ac.cn/gsa (accessed on 13 February 2026).

## 3. Results

### 3.1. Changes in Crude Polysaccharide Content Under Low-Temperature Storage

**Figure 1:**
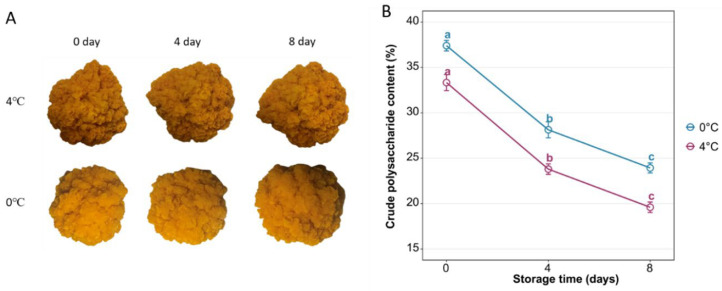
Morphology (**A**) and crude polysaccharide content (**B**) of JinEr mushroom fruiting bodies under different temperature and storage day treatments. Different letters (a–c) indicate significant differences at *p* < 0.05.

During the entire storage period, the fruiting bodies of JinEr mushroom exhibited relatively slight phenotypic variations (Figure 1A). Notably, no visible freezing injury or tissue necrosis was observed in any sample throughout storage, confirming that 0 °C constitutes moderate, non-injurious cold stress rather than freezing damage for the JinEr symbiotic complex. Storage temperature and storage duration significantly affected the crude polysaccharide content of JinEr mushroom (Figure 1B). Under both 0 °C and 4 °C conditions, the crude polysaccharide content showed a marked decreasing trend with prolonged storage—starting from an average of 37.40% (0 °C) and 33.33% (4 °C) in the OS group (Day 0), it declined to 28.13% (0 °C) vs. 23.80% (4 °C) at 4 days and 23.93% (0 °C) vs. 19.60% (4 °C) at 8 days—and the content at 0 °C was consistently higher than that at 4 °C at the same storage stage. There were significant differences in crude polysaccharide content among different storage days under the same temperature (*p* < 0.05), with the highest content in the OS group and the lowest seen at 8 days in both groups. Notably, the reduction was more pronounced in the 4 °C group (total reduction of ~41.2% vs. ~36.0% at 0 °C), suggesting more severe crude polysaccharide degradation at this temperature.

### 3.2. Global Metabolomic Shifts Reveal Temperature-Dependent Metabolic Variation

**Figure 2:**
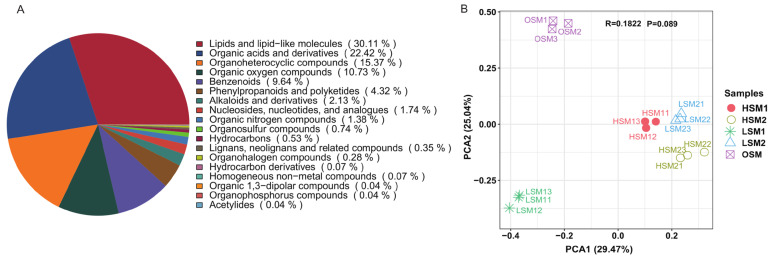
Non-targeted metabolomic analysis of JinEr mushroom. (**A**) Classification of all identified metabolites. (**B**) Principal component analysis (PCA) of the non-targeted metabolomics profiles from JinEr mushroom under different storage conditions.

**Figure 3:**
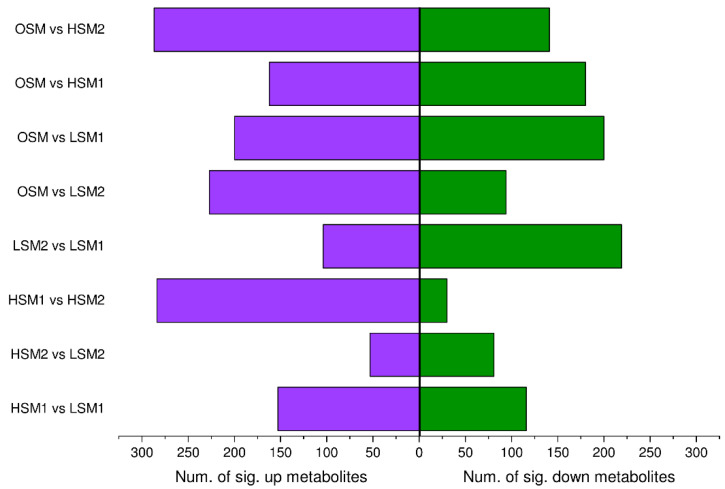
Comparison of the number of upregulated and downregulated differentially accumulated metabolites (DAMs) among all pairwise comparisons of five JinEr mushroom groups under low-temperature stress. Fold change (FC) = Group A/Group B. FC > 1.5 for upregulation. FC < 0.67 for downregulation. VIP > 1. *p*_adj_ < 0.05.

Non-targeted metabolomic profiling identified 2852 metabolites in JinEr fruiting bodies, with lipids and lipid-like molecules representing the most abundant class (Figure 2A). PCA of the metabolome revealed a clear separation of samples, driven primarily by storage duration and, to a lesser extent, by temperature (Figure 2B). This indicates that the metabolic state of the JinEr mushroom complex undergoes dynamic adjustment in response to postharvest storage conditions. Metabolomic analysis revealed that storage temperature critically shaped the metabolic adjustment of JinEr mushrooms over time (Figure 3, Table S1).

**Figure 4:**
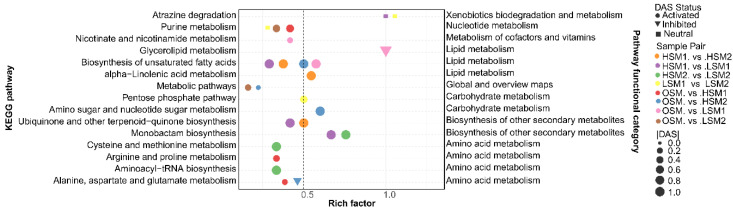
Metabolomic KEGG pathway enrichment analysis of intergroup differentially accumulated metabolites (DAMs) in JinEr mushroom under low-temperature stress. Only the significantly enriched pathways (*p*_adj_ < 0.05) are displayed.

Metabolic adjustments under different low temperatures showed divergent tendencies. At 0 °C, the metabolic response was dominated by pathways related to survival-essential functions. Membrane lipid remodeling-related pathways were prominently activated, exemplified by the biosynthesis of unsaturated fatty acids (OSM vs. LSM1, DAS = 1), while non-core metabolic pathways were inhibited—glycerolipid metabolism was specifically downregulated in short-term 0 °C storage (OSM vs. LSM1, DAS = −1) (Figure 4, Table S1). Additionally, the pentose phosphate pathway underwent notable remodeling in late-stage 0 °C storage (LSM1 vs. LSM2, DAS = 0.5), potentially supporting cellular redox homeostasis. At 4 °C, the metabolic pattern was more inclined to the maintenance of metabolic homeostasis. Basic metabolic pathways remained continuously activated, such as amino sugar and nucleotide sugar metabolism (OSM vs. HSM2, DAS = 1) and alanine, aspartate and glutamate metabolism (OSM vs. HSM1, DAS = 0.2) (Figure 4, Table S1). Alpha-linolenic acid metabolism (a lipid metabolism branch) was highly significantly enriched in short-term vs. long-term 4 °C storage (HSM1 vs. HSM2, DAS = 1). Secondary metabolism-related pathways were also enriched, including ubiquinone and other terpenoid-quinone biosynthesis (HSM1 vs. HSM2, DAS = 1) and monobactam biosynthesis (HSM2 vs. LSM2, DAS = 1), which complemented basic metabolic functions.

Across all low-temperature treatments, significantly activated pathways were concentrated in basic metabolism (amino acid, lipid, carbohydrate metabolism)—e.g., cysteine and methionine metabolism (HSM2 vs. LSM2, DAS = 1) and aminoacyl-tRNA biosynthesis (HSM2 vs. LSM2, DAS = 1) in cross-temperature comparisons, and biosynthesis of unsaturated fatty acids (consistently enriched, *p*_adj_ < 0.05) in lipid metabolism. Carbohydrate metabolism pathways (pentose phosphate pathway, amino sugar and nucleotide sugar metabolism) were also significantly regulated under specific conditions. In contrast, inhibited pathways were mainly non-core (e.g., glycerolipid metabolism, OSM vs. LSM1, DAS = −1). This pattern reflects a potential tendency of energy allocation adjustment under low-temperature stress (Figure 4, Table S1).

### 3.3. Transcriptomic Analysis of Low-Temperature Response

**Figure 5:**
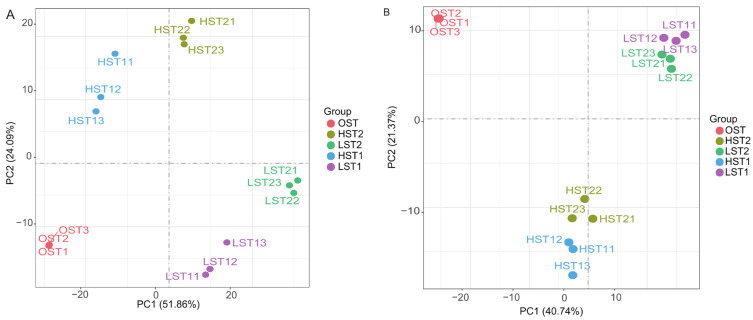
Principal component analysis (PCA) of JinEr mushroom transcriptomes under different storage conditions, aligned with the *N. aurantialba* (**A**) and *S. hirsutum* (**B**) transcriptome annotation.

**Figure 6:**
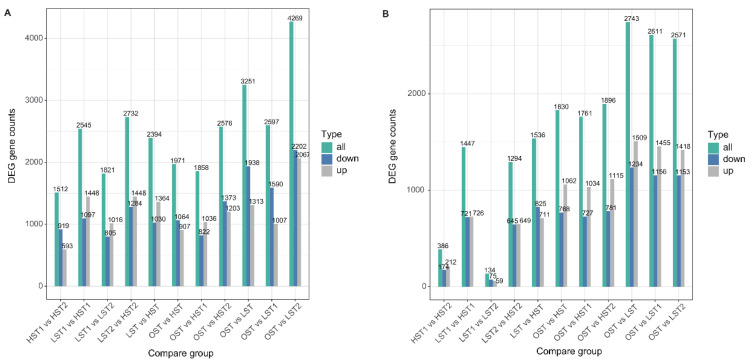
Number of shared and unique differentially expressed genes (DEGs) among sample groups in transcriptome analysis. (**A**) DEGs identified with *N. aurantialba* transcriptome annotation as a reference. (**B**) DEGs identified with *S. hirsutum* transcriptome annotation as a reference. DEGs were screened using the following criteria: |log2FC| > 1 and *p*_adj_ < 0.05.

Transcriptomic PCA showed that temperature was the primary driver of sample separation. This pattern was more distinct when aligned with the *S. hirsutum* genome, indicating a more temperature-specific transcriptional response in this fungus (Figure 5). While this reference yielded a higher number of constitutively expressed genes (Figure 6), alignment with the *N. aurantialba* genome identified a substantially larger pool of DEGs under stress. This response was most extreme in the LST2 vs. OST comparison (2743 DEGs) and least extreme in the HST1 vs. OST comparison (896 DEGs).

#### 3.3.1. *N. aurantialba*: Predominance of Stress Defense-Related Pathways

**Figure 7:**
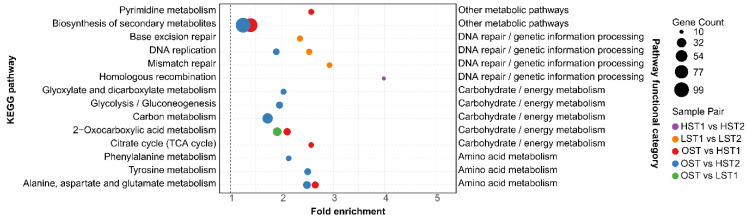
KEGG pathway enrichment analysis of differentially expressed genes (DEGs) between sample groups, using the *N. aurantialba* transcriptome annotation as a reference. Only the significantly enriched pathways (*p*_adj_ < 0.05) are displayed.

KEGG enrichment analysis of the *N. aurantialba* transcriptome revealed that its transcriptional response is distinctly characterized by the predominance of genetic information maintenance (Figure 7). Pathways involved in DNA repair and genetic information homeostasis maintenance constitute the transcriptional core of the low-temperature response in *N. aurantialba*. These pathways exhibit high enrichment significance and show coordinated functional features and distributions. They cover key processes such as DNA damage repair and replication fidelity regulation, forming a functionally linked network related to genomic stability. Representative pathways include homologous recombination (which is responsible for repairing DNA double-strand breaks), mismatch repair (which corrects base mismatches), and DNA replication (which regulates genomic replication processes) (Figure 7, Table S2). In terms of group distribution, these pathways display clear enrichment specificity. Homologous recombination is significantly enriched in comparisons of short-term storage at 4 °C vs. long-term storage (HSM1 vs. HSM2). Mismatch repair and DNA replication are significantly enriched in comparisons of short-term storage at 0 °C vs. long-term storage (LSM1 vs. LSM2). Certain pathways (e.g., DNA replication) also appear in comparisons of ambient temperature vs. long-term storage at 4 °C (OST vs. HSM2) (Figure 7, Table S2).

By contrast, metabolism-related pathways in *N. aurantialba* lack core transcriptional response characteristics and instead only show condition-specific transcriptional regulation patterns with limited coverage across the experimental treatments. In terms of coverage, their significant enrichment does not span the full range of experimental comparisons, encompassing 0 °C vs. 4 °C temperature gradients and short-term vs. long-term storage stages. This results in an absence of both a unified response network and functional consistency across different treatments. For example, amino acid metabolism pathways (e.g., alanine, aspartate and glutamate metabolism; tyrosine metabolism) are only enriched in 4 °C-related comparisons (OST vs. HSM1 and OST vs. HSM2), with no significant signals detected in any 0 °C-related comparisons. Meanwhile, carbohydrate metabolism pathways (e.g., the tricarboxylic acid cycle and 2-oxocarboxylic acid metabolism) are primarily restricted to ambient temperature versus short-term low-temperature comparisons (OST versus HSM1 and OST versus LSM1), with no enrichment observed in short-term versus long-term low-temperature comparisons. These metabolism-related pathways only exhibit transcriptional adjustments under specific local conditions, forming a sharp contrast with the systematic core response of genetic information maintenance pathways (Figure 7, Table S2).

#### 3.3.2. *S. hirsutum*: Predominance of Basal Metabolic Pathways

**Figure 8:**
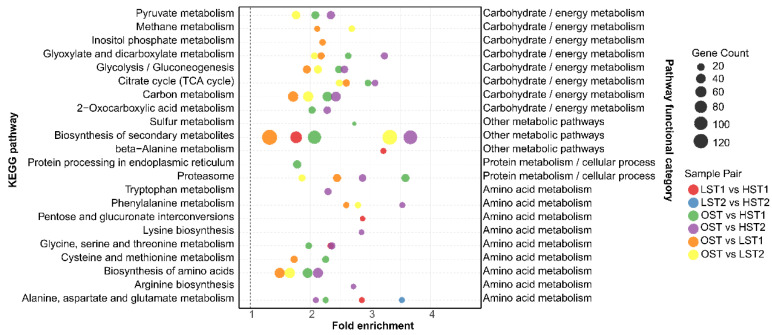
KEGG pathway enrichment analysis of differentially expressed genes (DEGs) between sample groups, using the *S. hirsutum* transcriptome annotation as a reference. Only the significantly enriched pathways (*p*_adj_ < 0.05) are displayed.

Transcriptomic KEGG pathway enrichment analysis (*p*_adj_ < 0.05) revealed that the transcriptional response of *S. hirsutum* to low temperatures is primarily driven by basal metabolic maintenance and protein homeostasis. There was no significant enrichment of stress defense-related pathways (e.g., DNA repair). This suggests that the low-temperature response of *S. hirsutum* showed a feature of an emphasis on metabolic maintenance and protein functional stability. Pathway enrichment exhibits global consistency and comprehensive coverage across all low-temperature conditions (Figure 8, Table S3). Basal metabolic pathways were significantly enriched in all comparative groups, including ‘control vs. low temperature’ (OST vs. HST1, OST vs. HST2, OST vs. LST1 and OST vs. LST2) and ‘short-term vs. long-term low temperature’ (HST1 vs. HST2 and LST1 vs. LST2). These pathways cover core metabolic branches, such as carbohydrate and amino acid metabolism. This reflects the core role and universality of basal metabolism in the low-temperature response of *S. hirsutum*. Concurrently, protein metabolism and homeostasis-related pathways exhibited highly significant enrichment in each key comparative group: ‘control vs. 4 °C’ (OST vs. HST1, OST vs. HST2), ‘control vs. 0 °C’ (OST vs. LST1, OST vs. LST2) and ‘short-term vs. long-term low temperature’ (HST1 vs. HST2, LST1 vs. LST2). These pathways emerged amongst the most representative features of the low-temperature transcriptional response in *S. hirsutum* (Figure 8, Table S3).

#### 3.3.3. Integrated Analysis of Gene Expression and Metabolic Changes in Key Pathways of *N. aurantialba* and *S. hirsutum*

**Figure 9:**
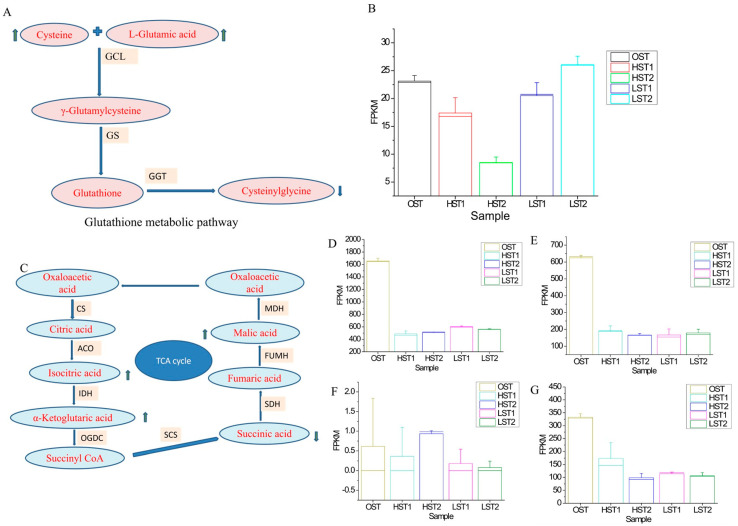
Integrated analysis of glutathione and TCA cycle pathways under low-temperature storage. (**A**) Glutathione metabolic pathway. (**B**) Expression profile of the glutathione synthase gene (*Gss*). Panels (**A**,**B**) are based on *N. aurantialba* transcriptome annotation. (**C**) TCA cycle. (**D**) Aconitase gene (Aco). (**E**) Isocitrate dehydrogenase gene (*Idh*). (**F**) Succinate dehydrogenase gene (*Sdh*). (**G**) Malate dehydrogenase gene (*Mdh*). Panels (**C**–**G**) are based on *S. hirsutum* transcriptome annotation. The upward blue arrow indicates upregulation (relative to the OSM sample group), and the downward blue arrow indicates downregulation.

Analysis of annotated metabolic pathways revealed that in the TCA cycle based on *S. hirsutum* annotation, isocitric acid, α-ketoglutaric acid, and malic acid were upregulated relative to the OST control, whereas succinic acid was downregulated. However, the expression of key TCA cycle genes, such as aconitase gene (*Aco*), isocitrate dehydrogenase gene (*Idh*) and succinate dehydrogenase gene (*Sdh*), was found to be lower than that in the OST control (Figure 9). In the glutathione pathway based on *N. aurantialba* annotation, cysteine and L-glutamic acid were found to be upregulated relative to the OST control, while cysteinylglycine was found to be downregulated. At the gene level, glutathione synthase gene (*Gss*) was upregulated in the LST2 group but downregulated in other sample groups compared to the OST control (Figure 9).

**Figure 10:**
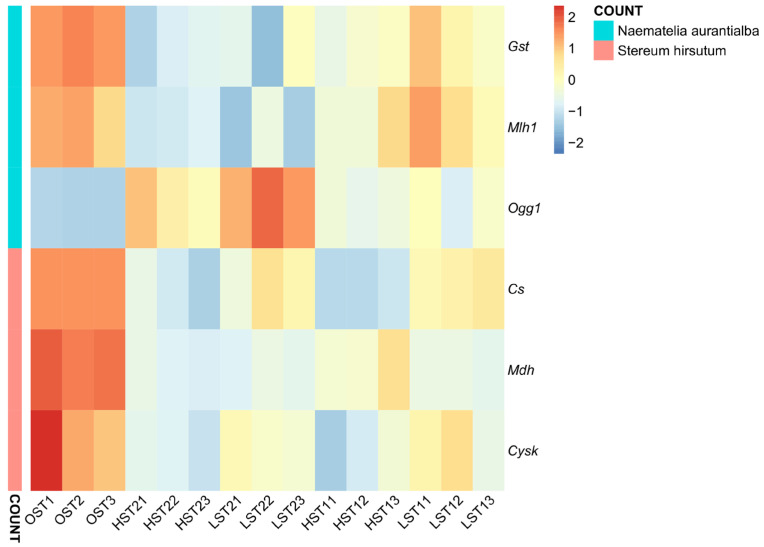
Gene expression heatmap of selected genes in sample groups based on fragments per kilobase of transcript per million mapped reads (FPKM) values (with FPKM transformation) in sample. *Gst*: glutathione S-transferase gene; *Mlh1*: MutL homolog 1 gene; *Ogg1*: 8-oxoguanine DNA glycosylase gene; *Cs*: citrate synthase gene; *Mdh*: malate dehydrogenase gene; *Cysk*: cysteine synthase gene.

Reflecting the differential response patterns described above, the expression patterns of key genes (quantified as FPKM) obviously differed between *N. aurantialba* and *S. hirsutum* under different storage conditions. For *N. aurantialba*, stress defense-related genes (*Gst*, *Mlh1* and *Ogg1*) exhibited divergent trends in response to low temperatures. *Gst* and *Mlh1* were relatively highly expressed in the OST control and LST1 (0 °C, 4 days) groups compared to the long-term cold storage groups (HST2: 4 °C, 8 days; LST2: 0 °C, 8 days) (Figure 10, Table S4). In contrast, *Ogg1* (a core gene for base excision repair) exhibited dramatically elevated expression in all cold-stressed groups relative to the OST group, consistent with its known role in base excision repair under cold stress (Table S4). For *S. hirsutum*, primary metabolism-related genes (*Cs*, *Mdh* and *Cysk*) displayed the highest expression in the OST control (Figure 10). Their expression was uniformly downregulated under low-temperature storage (with the lowest expression in HST2 and moderate reductions in LST1 and HST1).

### 3.4. Integrated Transcriptome-Metabolome Correlation Analysis

**Figure 11:**
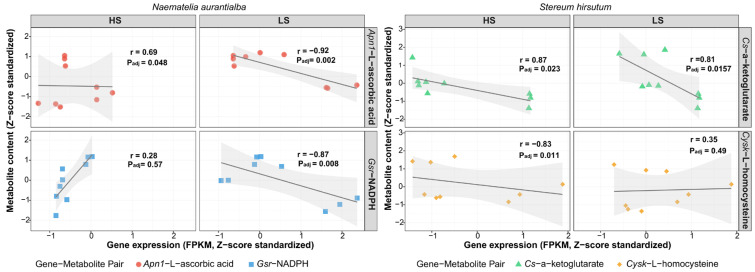
Spearman correlation map of key DEGs and DAMs in JinEr mushroom under different temperature regimes (HS: 4 °C; LS: 0 °C) with correlation coefficients displayed. *Apn1*: Aminopeptidase N1 gene. *Gsr*: Glutathione reductase gene.

Analysis based on the *N. aurantialba* transcriptome revealed a positive correlation between the base excision repair gene aminopeptidase N1 (*Apn1*) and L-ascorbic acid. This correlation strengthened from moderate at HS (4 °C storage; r = 0.69, *p*_adj_ = 0.048) to strong at LS (0 °C storage; r = 0.92, *p*_adj_ = 0.002) (Figure 11). Similarly, the glutathione metabolism gene glutathione reductase (*Gsr*) developed a strong negative correlation with NADPH specifically under LS conditions (r = −0.87, *p*_adj_ = 0.008). In contrast, analysis based on the *S. hirsutum* transcriptome showed a different pattern. The TCA cycle gene *Cs* maintained a stable positive correlation with α-ketoglutarate across both temperature conditions (HS: r = 0.78, *p*_adj_ = 0.023; LS: r = 0.81, *p*_adj_ = 0.015). The cysteine synthesis gene *Cysk* showed a strong negative correlation with L-homocysteine at HS (r = −0.83, *p*_adj_ = 0.011), a correlation which disappeared at LS (r = 0.35, *p*_adj_ = 0.49).

### 3.5. Enzyme Activity Validation

**Figure 12:**
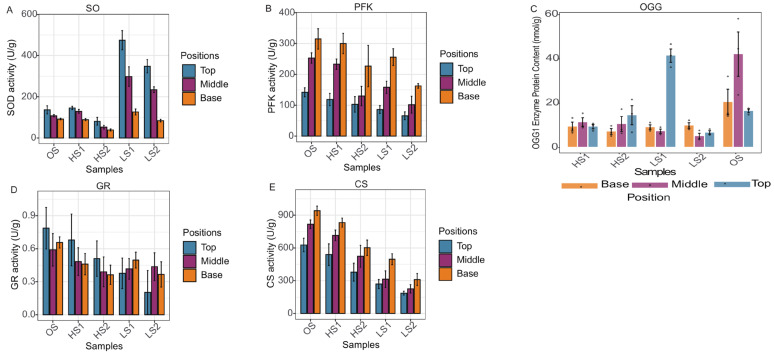
Result of activities of five key enzymes in different storage treatment groups of JinEr mushroom with analysis of apical/middle/basal regions. (**A**) Superoxide dismutase (SOD); (**B**) phosphofructokinase (PFK); (**C**) 8-oxoguanine DNA glycosylase 1 (OGG1); (**D**) glutathione reductase (GR); (**E**) citrate synthase (CS).

Enzyme activity assays in JinEr mushroom fruiting bodies revealed distinct spatial gradients. SOD activity followed a “T > M > B” pattern. In the LS1 group, apical SOD activity (152.1 U/g) was significantly higher than in the middle (112.3 U/g, *p*_adj_ = 0.028) and basal (89.5 U/g, *p*_adj_ = 0.016) regions, while apical SOD in HS1 was notably lower than in LS1 (*p*_adj_ = 0.016) (Figure 12). GR activity was also highest in apical regions, with LS1 apical GR significantly exceeding basal levels (*p*_adj_ = 0.048), and HS2 apical GR activity was significantly higher than LS2 apical activity (*p*_adj_ = 0.028).

CS exhibited a “B > M > T” gradient. In HS1, basal CS activity (886.5 U/g) was significantly higher than in middle (672.3, *p*_adj_ = 0.042) and apical regions (518.2, *p*_adj_ = 0.028). Similarly, basal CS in the OS group exceeded all other regions (all *p*_adj_ < 0.05) (Figure 12). PFK activity was elevated basally, with LS1 basal PFK being significantly higher than apical (*p*_adj_ = 0.028), and HS1 basal PFK surpassing LS1 basal levels (*p*_adj_ = 0.028). OGG1 content showed spatial and treatment-related variations (*p*_adj_ < 0.05). The OS group had the highest OGG1 in the middle region (41.8 nmol/g), significantly above its own top (16.2 nmol/g) and base (20.2 nmol/g) regions and middle regions of other groups. LS1 showed the highest OGG1 in the top region, significantly exceeding top regions of other groups. No significant differences were found in the basal region across groups.

## 4. Discussion

This study integrated transcriptomics, spatially resolved enzymatic analysis, and metabolomics to reveal distinct low-temperature stress response patterns between the two symbiotic fungi comprising the JinEr mushroom fruiting body. These differential responses, observed through correlative multi-omics and biochemical assays, align with previously reported species-specific spatial distributions within the fruiting body [4]. Based on these findings, we propose a working model in which the two symbionts exhibit context-dependent stress response tendencies under the studied conditions. This model, while consistent with our multi-omics and enzymatic data, requires direct functional validation (e.g., gene knockout or complementary culture experiments) to be conclusively established.

Transcriptome analysis of the *N. aurantialba* genome in this study has demonstrated that this fungus exhibits a pronounced response oriented towards stress defense at the transcriptional and enzymatic levels. The DEGs were significantly enriched in pathways such as DNA damage repair and glutathione metabolism, and key genes such as *Ogg1* and *Gst* were significantly upregulated. Correspondingly, the SOD and GR enzyme activities in the top region of the fruiting body (enriched in *N. aurantialba*) were significantly higher. This spatial gradient of enzyme activity is consistent with the species-specific distribution pattern reported by Lan et al. [4]: the apical region of the JinEr fruiting body has the most balanced ratio of *S. hirsutum* to *N. aurantialba* (1.51:1), with *N. aurantialba* accounting for a relatively higher proportion compared to the middle (3.97:1) and basal (6.52:1) regions. Conversely, *N. aurantialba* has been reported as a key species driving the formation of special gelatinous fruiting bodies in the JinEr mushroom, with an ecological niche that is more inclined to obtain nutrients from symbiotic partners to complete morphological construction [12,13,14]. Our multi-omics and biochemical data indicate that *N. aurantialba* showed a pronounced response bias toward stress defense pathways during postharvest cold storage, which likely helps preserve cellular and structural integrity of the symbiotic complex.

In contrast, the transcriptomic response of *S. hirsutum* was dominated by fundamental metabolic pathways, including carbon metabolism, the TCA cycle, and amino acid biosynthesis, with little enrichment of canonical stress defense pathways. Concurrently, the basal region, where *S. hirsutum* is predominantly distributed, exhibited relatively high activities of metabolic enzymes such as CS and PFK. These patterns align with the findings of Lan et al. [4], who reported that *S. hirsutum* dominates the basal region and substrate (representing 77.12% of the total genotype content in the fruiting body) and acts as a major contributor to substrate decomposition and nutrient acquisition within the symbiotic system. Previous studies have confirmed that *S. hirsutum* is the only functional fungus in the JinEr mushroom cultivation system that can effectively colonize and degrade lignocellulosic substrates, playing a “cornerstone” role in terms of providing initial nutrition to symbiotic organisms [14,15]. The present study suggests that, in response to postharvest low-temperature stress, *S. hirsutum* exhibited a sustained transcriptional and enzymatic orientation toward basal metabolic homeostasis and potentially contributed to the overall stability of the symbiotic system. Although transcript levels and enzyme activities generally followed consistent functional trends, some divergence may arise from post-transcriptional regulation or protein stability, rather than a direct one-to-one relationship between gene expression and enzyme activity.

This study observed an apparent contradiction in both *S. hirsutum* and *N. aurantialba* under postharvest low-temperature stress: the downregulation of partial core genes coexisted with the preservation of the pathways’ primary biological functions. For *S. hirsutum* although several core TCA cycle genes were downregulated at the transcriptional level, the activities of key metabolic enzymes including CS and PFK remained consistently high in the basal region where this fungus was predominantly distributed (Figure 12), and critical metabolites in the TCA pathway such as isocitric acid and α-ketoglutaric acid were significantly upregulated (Figure 9). These lines of evidence directly confirm the maintenance of TCA pathway function despite the downregulation of individual core genes. Similar phenomena have been reported in other edible fungi, attributed to post-transcriptional regulation, compensatory enzyme activity, metabolic flux redistribution, and pathway redundancy [16,17]. In the JinEr mushroom symbiosis, KEGG enrichment analysis confirmed that central metabolic pathways in *S. hirsutum* and stress defense pathways in *N. aurantialba* were consistently enriched under cold stress (Figure 7 and Figure 8). Moreover, most genes within these core pathways were predominantly upregulated across all cold stress groups (Tables S2 and S3). In both fungi, individual gene downregulation likely represents fine regulatory tuning that does not block overall pathway flux, which may be sustained via post-transcriptional regulation and isoenzyme compensation [16,17]. Thus, the observed divergent responses are context-specific adaptations to postharvest cold stress, with the two fungi maintaining the activity of their respective core functional pathways at the systemic level. However, this study lacks direct experimental evidence to verify these potential regulatory mechanisms, which remain to be further elucidated in future research.

A well-known example of fungal symbiosis in edible mushrooms is the YinEr mushroom (*Tremella fuciformis*), whose fruiting body formation depends on its interaction with the companion fungus *Annulohypoxylon stygium* [16,17,18]. The YinEr fruiting body is homogeneous, being formed exclusively by *T. fuciformis* [19,20,21]. In this system, functional partitioning between the two fungi occurs during the cultivation stage. *T. fuciformis* lacks the enzymes required for lignocellulose degradation and thus relies on *A. stygium* to break down wood substrates and provide accessible carbon sources, enabling its own fruiting body morphogenesis [19]. However, the fruiting body of the JinEr mushroom is composed of *N. aurantialba* and *S. hirsutum*. These two symbionts exhibited differential stress response patterns in the postharvest cold stress conditions investigated in this study. This different response may highlight a context-dependent divergence in stress responses between the two symbionts, distinct from the morphology–nutrition partitioning described in the YinEr system during cultivation. Further studies are needed to compare the symbiotic response mechanisms of these two systems across a broader range of environmental conditions.

This study found that the response of the JinEr mushroom complex differed markedly depending on storage temperature. At 0 °C, the metabolic and transcriptional profiles jointly pointed to a basic survival mode—characterized by the significant upregulation of unsaturated fatty acid biosynthesis (to maintain membrane fluidity) and accompanied by the inhibition of energy metabolism. This strategy is consistent with the common acute cold stress response mechanism in the biological world [22,23,24], and its core function is to ensure the basic structural integrity of cells [25,26,27]. At 4 °C, the system shifted to a more proactive maintenance and defense mode. Its characteristic was the activation of high-fidelity homologous recombination repair pathways and the biosynthesis of specific defense metabolites (e.g., ubiquinone). This distinction resembles the shift from rapid stress tolerance to a more regulated stress response state observed in many postharvest commodities [28,29], reflecting the graded cold stress response characteristics of the JinEr symbiotic complex to different levels of postharvest cold stress intensity [30,31].

It is important to note that the differential responses observed in this study are based on correlative multi-omics and spatial enzyme assays. While consistent with a possible functional bias under cold stress, these patterns do not yet establish a fixed division of labor. Instead, we present them as a context-specific response model that requires validation through targeted experiments, such as mono-culture stress tests or genetic interference studies.

## 5. Conclusions

This study revealed temperature-dependent differential response tendencies in the symbiotic fungi *N. aurantialba* and *S. hirsutum* under postharvest cold storage. Under the studied conditions, *N. aurantialba* showed enhanced stress defense responses (e.g., DNA repair, antioxidant systems), whereas *S. hirsutum* exhibited sustained basal metabolic homeostasis. This differential response pattern was associated with a slower polysaccharide loss at 0 °C and more pronounced polysaccharide loss at 4 °C. However, these findings are based on correlative multi-omics data with limited replicates and without direct functional validation, and therefore should be interpreted as a working model that requires further confirmation. From a practical perspective, 0 °C is recommended for long-term preservation and 4 °C is recommended for the short-term storage of JinEr mushrooms.

## Data Availability

Data will be made available on request.

## Supplementary Materials

The following supporting information can be downloaded at: https://www.mdpi.com/article/10.3390/genes17030296/s1, Table S1: Enrichment analysis of KEGG pathway in non-target metabolome. DAS: differential abundance score; Table S2: Enrichment analysis of KEGG pathway using *Naematelia aurantialba* transcriptome annotation; Table S3: Enrichment analysis of KEGG pathway using *Stereum hirsutum* transcriptome annotation; Table S4: log_2_FC and *p*_adj_ Values of Key DEGs annotated against *Naematelia aurantialba* and *Stereum hirsutum* Reference Genomes (Low-Temperature vs. Control).

## References

[1] Li C., Xu S. (2022). Edible mushroom industry in China: Current state and perspectives. Appl. Microbiol. Biotechnol..

[2] Li D.-M., Zhang S.-W., Sun J.-Y., Li Y.-J., Li W.-P. (2024). Analysis of amino acid content and nutritional value evaluation in Golden Ear from different origins. J. Food Saf. Food Qual..

[3] Wu R.-N., Zhu Y.-Y., Ma R.-H., Ni Z.-J., Deng X.-J., Thakur K., Wei Z.-J. (2025). Purification, structural characteristics, bioactive properties, and applications of *Naematelia aurantialba* polysaccharides: A comprehensive review. Molecules.

[4] Lan J., Zhang Y., Cai Y., Shi X., Zhang K., Huang J., Yang C., He X., Yu F., Liu W. (2025). Spatial ratio of two fungal genotypes content of *Naematelia aurantialba* and *Stereum hirsutum* in nutritional growth substrate and fruiting bodies reveals their potential parasitic life cycle characteristics. J. Agr. Food Res..

[5] Yang Y., Dong C. (2024). Fruiting body heterogeneity, eimorphism and haustorium-like structure of *Naematelia aurantialba* (JinEr Mushroom). J. Fungi.

[6] Suárez M., Sáez-Casado M., Martínez T., Rincόn-Cervera M. (2024). The effect of low temperature storage on the lipid quality of fish, either alone or combined with alternative preservation technologies. Foods.

[7] Yuan Q., Jiang Y., Yang Q., Li W., Gan G., Cai L., Li W., Qin C., Yu C., Wang Y. (2024). Mechanisms and control measures of low temperature storage-induced chilling injury to solanaceous vegetables and fruits. Front. Plant Sci..

[8] Zha L., Chen M., Yu C., Guo Q., Zhao X., Li Z., Zhao Y., Li C., Yang Y. (2020). Differential proteomics study of postharvest *Volvariella volvacea* during storage at 4 °C. Sci. Rep..

[9] Farcuh M. (2025). Understanding chilling injury and sugar metabolism-related genes and metabolites in ‘Red Haven’ peaches. Plants.

[10] Wang R., Hirabayashi M., Furuta A., Okazaki T., Tanimoto S. (2023). Changes in extractive components and bacterial flora in live mussels *Mytilus galloprovincialis* during storage at different temperatures. J. Food Sci..

[11] Hakimi A.A., Reznik E., Lee C.-H., Creighton C.J., Brannon A.R., Luna A., Aksoy B.A., Liu E.M., Shen R., Lee W. (2016). An integrated metabolic atlas of clear cell renal cell carcinoma. Cancer Cell.

[12] Cao Y., Yang L.-L., Li R.-C., Feng F.-J., Li M.-J., Luo X.-Y., Shen Z.-H., Lu Q.-Q. (2022). A preliminary study on composition of fungal species and nutrient transportation in *Naematelia aurantialba* culture. Acta Edul. Fungi.

[13] Cao Y., Shen Z.-H., Yang L.-L., Li M.-G., Luo X.-Y., Yang X.-J., Lu Q.-Q., Li R.-C. (2024). Determination of polarity of *Naematelia aurantialba* by single spore hybridization. Acta Edul. Fungi.

[14] Yang Y., Lu J., Dong C. (2025). Pheromone MAPK pathway regulates the yeast-to-hypha transition in the parasitic mushroom *Naematelia sinensis* in a cell fusion-independent manner. Microbiol. Res..

[15] Mišković J., Rašeta M., Čapelja E., Krsmanović N., Novaković A., Karaman M. (2021). Mushroom species *Stereum hirsutum* as natural source of phenolics and fatty acids as antioxidants and acetylcholinesterase inhibitors. Chem. Biodiver..

[16] Li D., Wang D., Fang Y., Li L., Lin X., Xu Y., Chen H., Zhu M., Luo Z. (2021). A novel phase change coolant promoted quality attributes and glutamate accumulation in postharvest shiitake mushrooms involved in energy metabolism. Food Chem..

[17] Cai Z., Zeng Z., Chen W., Guo Z., Zheng H., Lu Y., Zeng H., Chen M. (2025). Comprehensive metabolomic and transcriptomic analysis revealed the molecular basis of the effects of different refrigeration durations on the metabolism of *Agaricus bisporus* cultivation spawn. Curr. Tradit. Med..

[18] Deng Y., van Peer A.F., Lan F.-S., Wang Q.-F., Jiang Y., Lian L.-D., Lu D.-M., Xie B. (2016). Morphological and molecular analysis identifies the associated fungus (“Xianghui”) of the medicinal white jelly mushroom, *Tremella fuciformis*, as *Annulohypoxylon stygium*. Int. J. Med. Mushrooms.

[19] Liu D., Pujiana D., Wang Y., Zhang Z., Zheng L., Chen L., Ma A. (2019). Comparative transcriptomic analysis identified differentially expressed genes and pathways involved in the interaction between *Tremella fuciformis* and *Annulohypoxylon stygium*. Antonie Leeuwenh..

[20] Liu D., Sun X., Yan B., Ma A. (2022). Alternative oxidase is involved in oxidative stress resistance and melanin synthesis in *Annulohypoxylon stygium*, a companion fungus of *Tremella fuciformis*. Antonie Leeuwenh..

[21] Li Y., Cao J., Zhan G., Jia J., Fan J., Shen Z., Chen L., Sun S. (2025). Time-course proteomics analysis and gene function validation of promoting dimorphic transition of *Tremella fuciformis* by *Annulohypoxylon stygium* Extract. Food Biosci..

[22] Khakhar A. (2023). A roadmap for the creation of synthetic lichen. Biochem. Biophys. Res. Commun..

[23] Osyczka P., Lenart-Boroń A., Boroń P., Rola K. (2021). Lichen-forming fungi in postindustrial habitats involve alternative photobionts. Mycologia.

[24] Gao H., Ye S., Liu Y., Fan X., Yin C., Liu Y., Liu J., Qiao Y., Chen X., Yao F. (2023). Transcriptome analysis provides insight into gamma irradiation delaying quality deterioration of postharvest *Lentinula edodes* during cold storage. Food Chem..

[25] Liu Q., Cui X., Song Z., Kong W., Kang Y., Kong W., Ng T.B. (2021). Coating shiitake mushrooms (*Lentinus edodes*) with a polysaccharide from *Oudemansiella radicata* improves product quality and flavor during postharvest storage. Food Chem..

[26] Yang Y., Nian S., Yu J., Jing S., Zhu B., Wang K., Shi Y., Bai J., Xu H., Kou L. (2025). Exploring the possible mechanisms of X-Rays treatment for retention aroma volatiles in shiitake mushrooms during low temperature storage. Food Chem..

[27] Jiang W., Gu Y., Liu Y., Han T., Song Z., Zhu D., Li J., Cheng F. (2024). Effect of storage temperature on quality of *Oudemansiella raphanipies* and prediction model of shelf life. Shandong Agric. Sci..

[28] Ren Y., Ma Q., Li D., Chen Y., Cheng Q., Luo Z. (2025). Autophagy-mediated energy charge sustainability alleviated postharvest quality deterioration of strawberry (*Fragaria* × *Ananassa*). Food Chem..

[29] Zhang J., Wang C., Chen C., Zhang S., Zhao X., Wu C., Kou X., Xue Z. (2023). Glycine betaine inhibits postharvest softening and quality decline of winter jujube fruit by regulating energy and antioxidant metabolism. Food Chem..

[30] Sugita K., Takahashi S., Uemura M., Kawamura Y. (2024). Freezing treatment under light conditions leads to a dramatic enhancement of freezing tolerance in cold-acclimated Arabidopsis. Plant Cell Environ..

[31] Yang H., Qiao K.-W., Teng J.-J., Chen J.-B., Zhong Y.-L., Rao L.-Q., Xiong X.-Y., Li H. (2023). Protease inhibitor ASP enhances freezing tolerance by inhibiting protein degradation in kumquat. Hortic. Res..

